# Genital *Chlamydia trachomatis* Infection among Women of Reproductive Age Attending the Gynecology Clinic of Hawassa University Referral Hospital, Southern Ethiopia

**DOI:** 10.1371/journal.pone.0168580

**Published:** 2016-12-22

**Authors:** Endale Tadesse, Million Teshome, Anteneh Amsalu, Techalew Shimelis

**Affiliations:** 1 Department of Medical Laboratory Science, College of Medicine and Health Sciences, Hawassa University, Hawassa, Ethiopia; 2 Gynecology and Obstetrics Unit, College of Medicine and Health Sciences, Hawassa University, Hawassa, Ethiopia; University of Illinois at Urbana-Champaign, UNITED STATES

## Abstract

**Background:**

Urogenital infection with *Chlamydia trachomatis*(CT) is one of the most common bacterial sexually transmitted infections (STIs) world-wide, especially in developing nations where routine laboratory diagnosis is unavailable. Little is known about the epidemiology of this infection in Ethiopia where other STIs are prevalent. This study was conducted to determine the prevalence and associated factors of CT infection among women of reproductive age.

**Methods:**

A cross-sectional study was conducted among 322 consecutive women aged between 15–49 years at Hawassa University Referral Hospital from November 2014 to April 2015. Data on socio-demography and potential risk factors for genital infection were collected using structured questionnaires. Moreover, endocervical swabs were collected from all participants, screened for CT antigen using rapid immunochromatography assay, and cultured following the standard bacteriological method to isolate *Neisseria gonorrhoeae*.

**Result:**

In this study, the overall prevalence of CT antigen and *N*. *gonorrhoeae* infection was 61(18.9%) and 1(0.31%), respectively. Women aged 15–24 years had the highest prevalence of CT infection (24.2%), followed by those aged 25–34 years (16.8%) and those aged 35–49 years (9.6%). CTinfection was associated with women who had unprotected sex within the last six months (aOR = 3.459; 95% CI = 1.459–8.222) and were sexually active for 6–10 years (aOR = 3.076; 95% CI = 1.152–8.209). None of the clinical symptoms and diagnoses was significantly associated with CT antigen positivity.

**Conclusions:**

The high prevalence of genital CT infection in this study highlights the need for further large-scale studies on the general population. Thus, screening of women regardless of their symptoms should be in place.

## Background

Sexually transmitted infections (STIs) are major public health problems in developing countries where diagnostic and treatment service is limited [1]. *Chlamydia trachomatis*(CT) and *Neisseria gonorrhoeae* are the most common causes of genitourinary tract infection in young adults around the world [2]. Globally, over 90 million new CT infections were reported annually [3]. CT infection is known to cause cervicitis and non-gonococcal urethritis. Women with cervical chlamydial infection are at a risk for pelvic inflammatory disease (PID), which can lead to long—term reproductive sequelae, such aschronic pelvic pain, ectopic pregnancy and infertility [4]. However, about 75% of women with CT infection remain asymptomatic and because they are unaware of their infection, they do not seek treatment [5]. Consequently, unrecognized and untreated individuals remain infectious for months during which there is an increased chance of transmitting the pathogen to their sexual partners.

As part of an effort to reduce the transmission and clinical consequences of chlamydial infection, the Center for Disease Control and Prevention (CDC) recommends annual screening of all sexually active women aged less than 25 years [6], and study has shown thatannual screening would prevent 61% of CT-related PID in women who became infected with CT [7]. However, the current practice in Ethiopia is based on syndromic case management by adopting the world health organization(WHO) recommendation, despite its poor specificity and positive predictive values [1].

In Ethiopia, the impact of CT infection is not well known due to the asymptomatic nature of the infection and lack of laboratory diagnostic facilities. In a previous study, the sero-prevalence of genital chlamydial infection among Ethiopian women was shown to be 62%with42% havingtiters suggestive of recent or present genital infection. In the same study, factors found to be associated with the infection were age at first coitus, religion, prostitution, number of sexual partners and present age of the woman [8]. However, further investigations on the epidemiology of genital chlamydial infection in Ethiopiaare required to understand whether the country’s comprehensive and expanded human immunodeficiency virus (HIV)responseshave impacted the significance of CT infection. This study wasconducted to generateevidence oncurrent prevalence and associated risk factors of genital chlamydial infection among women of reproductive agein order to inform decision makers, service providers and other concerned bodies for possible interventions.

## Methods

### Ethical clearance

The study was ethically approved by the Institutional Review Board of College of Medicine and Health Sciences, Hawassa University. Participation was fully voluntary and informed written consent was obtained from all participants prior to their participation in the study. Parents/guardians gave verbally informed assentfor patients aged 15–17 years. Patient information was anonymized and de-identified prior to analysis. Screening of participants for genital CTand *N*. *gonorrhoeae* infections was performed free of charge and those found positive were managed by a physician. Any information obtained during the study was kept confidential.

### Study design, area and period

A cross-sectional study was carried out to investigate women attending the gynecology clinic of Hawassa University Referral Hospital in southern Ethiopia from November 2014 to April 2015. The hospital is located at Hawassa, the Capital City of the Southern Nations, Nationalities and Peoples’ Region (SNNPR). The hospital (with 400-bed capacity)is the largest in the regional state and provides health care for large numbers of patients.

### Study population

A consecutive group of 322 women aged between 15 and 49 years with or without symptoms of STIs and attending the Gynecology clinic for family planning, infertility examination or diagnosis and treatment services of STIs was enrolled. Women who were pregnant, had taken antibiotics within the last four weeks or had abnormal bleeding at the time of diagnosis were excluded from the study.

### Data collection

#### Socio demographic and clinical data collection

After obtaining informed written consent from the study participants, two trained nurses collected data on socio-demography, sexual history, behavioral factors and clinical symptoms using structured questionnaires. Subsequently, a gynecologist did a genital examination and then the clinical diagnoses were collected.

#### Sample collection and testing

Two endocervical swab specimens were collected aseptically from each participant; one for gonococcal culture, and one for CT antigen testing by a gynecologist using two Dacron-tipped swabs individually. A swab forrapid CT antigen test was performed immediately at the clinic; and a second swab, for isolation of *N*. *gonorrhoeae*, was placed into a capped tube and delivered to the laboratory immediately.

#### Isolation of *N*. *gonorrhoeae*

The swab samples received in the laboratory were inoculated into the Modified Thayer Martin agar, placed in an anaerobic jar with 5–10% CO_2_, and incubated at 37°C. Bacterial growth was checked every 24 hours for 72 hours. Bacterial isolates were identified based on their characteristic appearance, Gram reaction and pattern of biochemical reactions [9]. A reference strain of *N*. *gonorrhoeae*(ATCC 49226) was tested as a control.

#### Testing for CT antigen

CTantigen was detected by a laboratory technologist in the gynecology clinicusing the SD BIOLINE rapid antigen test kit (Standard Diagnostic Inc., Korea). Thisis a solid phase immunochromatographic assay for the rapid, qualitative detection of CTantigen directly from an endocervical swab. Tests were performed according to the instructions of the manufacturer. Briefly, to extract *Chlamydia* antigen, a swab was inserted in a tube containing 300 μL reagent A (sample digesting reagent) and incubated for 2 minutes. After mixing, 600μl of reagent B was added and mixed by rotating the swab 10 times. Then, the swab was discarded from the tube and the tube was assembled by dropping cap. Four drops of this extract was added into the sample well. The result was read at 15 min. A positive test result wasindicated when control (C) and test line (T) were visible. Anegative result was marked only by a visible control line(C). In case the control line did not appear, the result was interpreted as invalid and the test was repeated. The sensitivity and specificity of the test kit was 93.1% and 98.8%, respectively [10]. Samples positive for CT antigen were re-tested asecond time by the same method.

### Operational definitions

#### Unprotected sex

Study participants who practiced sex without condom use in the previous 6 months.

#### Urethritis cases

Patients reporting either dysuria and/or vaginal discharge at the time of diagnoses.

#### Data analysis

Data entry and analysis were performed using Statistical Package for Social Science (SPSS) version-16, and results are summarized using descriptive statistics. Multivariable logistic regression analysis was performed by taking those socio-demographic and risk factors found to be significantly associated with chlamydial infection in bivariate logistic regression analysis. Odds ratio (OR) with 95% confidence interval was used to measure the degree of association between dependent and independent variables. A p-value < 0.05 was considered to be statistically significant.

## Results

### Participant characteristics

A total of 329 women of reproductive age group attending the gynecology clinic were approached during the study period. Of these, seven (2.1%) refused to consent and were excluded. Thus, data from 322 women was considered for analysis. The majority (77.3%) of the study participants were married with a mean age of 29.6 years (standard deviation (SD), 8.2; range, 15–49 years). Among the women who participated, 28.3% were between 15–24 years of age, 39.4% were between 25–34 years of age and 32.3% were between 35–49 years of age. Concerning the educational status and residence, 34.2% were illiterate and 53.7% were rural dwellers. Among the study participants, 69.3% had one lifetime partner and 92.5% practiced unprotected sex within the last 6 months. The majority (70.2%) of study participants had no history of pre-marital sex and had not ever used any type of contraceptive methods (Table 1).

**Table 1:** Distribution of *Chlamydia trachomatis* infection by socio-demographic characteristic of women attending the gynecology clinic of Hawassa University Referral Hospital, 2015.

Variable	ParticipantsN (%)	CT Pos. N (%)	COR(95%CI)	p-value	aOR(95%CI)	p- value
**Age group(years)**						
15–24	91(28.3)	22(24.2)	2.997(1.334–6.734)	0.008*	1.716(0.603–4.879)	0.312
25–34	127(39.4)	29(22.8)	2.782(1.285–6.022)	0.009*	1.741(0.696–4.358)	0.236
35–49	104(32.3)	10(9.6)	1		1	
**Residence**						
Rural	173(53.7)	34(19.7)	1.105(0.631–1.936)	0.726	-	-
Urban	149(46.3)	27(18.1)	1			
**Marital status**						
Married	249(77.3)	45(18.1)	2.426(0.305–19.276)	0.402	-	-
Single	48(14.9)	12(25.0)	3.667(0.428–31.441)	0.236	-	-
Divorced	13(4.0)	3(23.1)	3.300(0.294–37.103	0.334	-	-
Widowed	12(3.7)	1(8.3)	1			
**Education**						
Illiterate	101(34.2)	14(13.9)	1			
1–6 grade	72(22.4)	17(23.6)	2.119(0.970–4.629)	0.059	-	-
7–12 grade	64(19.9)	13(20.3)	1.748(0.764–4.000)	0.186	-	-
>12 grade	76(23.6)	17(22.4)	1.976(0.907–4.302)	0.086	-	-
**Life time partner**						
0	24(7.3)	8(33.3)	2.434(0.972–6.094)	0.057	-	-
1	223(69.3)	38(17.0)	1		-	-
≥2	75(23.3)	15(20.0)	1.217(0.626–2.366)	0.52	-	-
**Unprotected sex within the last 6 months**						
Yes	24(7.5)	10(41.7)	3.459(1.459–8.222)	0.005*	3.314(1.334–8.232)	0.010*
No	298(92.5)	51(17.1)	1		1	
**Number of sexually active years**						
<1	57(17.7)	14(24.6)	3.437(1.381–8.551)	0.008*	2.149(0.688–6.716)	0.188
1–5	97(30.1)	21(21.6)	2.917((1.263–6.737)	0.012*	2.931(0.751–5.634)	0.161
6–10	64(19.9)	17(26.6)	3.818(1.583–9.208)	0.003*	3.076(1.152–8.209)	0.025
>10	104(32.3)	9(8.7)	1		1	
**Use of contraceptive**						
Yes	96(29.8)	20(20.8)	1.187(0.653–2.158)	0.573		-
No	226(70.2)	41(18.1)	1			-
**Type of contraceptive**						
Pills	10(10.4)	1(10.0)	1			
IUCD	11(11.5)	1(9.1)	0.9(0.049–16.594)	0.944	-	-
Implant	26(27.1)	6(23.7)	2.700(0.282–25.834)	0.389	-	-
Injectable	49(51.0)	12(24.5)	2.919(0.335–25.467)	0.335	-	-
**Pre-marital sex**						
Yes	96(29.8)	20(20.8)	1.187(0.653–2.158)	0.573	-	-
No	226(70.2)	41(18.1)	1			

* Significant association;

CT, *Chlamydia trachomatis*; Pos, positive; CI, confidence interval; N, total number; COR, crude odds ratio; aOR, adjusted odds ratio; 1–6 grade, Elementary school; 7–12 grade, Junior and high school; >12 grade, College and University; IUCD, intrauterine contraceptive device.

### Prevalence and associated risk factors

The overall prevalence was 18.9% (61/322) for CT and 0.31% (1/322) for *N*. *gonorrhoeae*. Women aged 15–24 years had the highest prevalence of CT infection (24.2%) followed by those aged 25–34 years (16.8%) and those aged 35–49 years (9.6%)(p<0.05).

In bivariate analysis, the prevalence of CT significantly decreased as age increased: women aged 15–24 and 25–34years were almost 3 times (COR = 2.997; 95% CI 1.334–6.734; p = 0.008) and 2.7 times (COR = 2.782; 95% CI 1.285–6.022; p = 0.009) more likely infected with CT, respectively, as compared to women aged 35–49 years. Women having unprotected sex (no condom use) within the last 6 months were about 3.5 times (COR = 3.459, 95% CI: 1.459–8.222; p = 0.005) more likely infected with CT as compared to those who used condoms. Women who weresexually active for less than a year (COR = 3.437, 95% CI: 1.381–8.551; p = 0.008) or for 1–5 years (COR = 2.917; 95% CI 1.263–6.737; p = 0.012) or for 6–10 years (COR = 3.818; 95% CI 1.583–9.208; p = 0.003) had higher odds of CT infection as compared to those who were sexually active for more than ten years. Other factors such as education, residence, marital status, number of lifetime partners, contraceptive usage, and history of pre-marital sex were not found to be associated with CT infection (Table 1).

In further analysis, after adjustment for those significantly associated variables using multivariable logistic regression, the risk of having CT remained significantly higher in women who had unprotected sex within the last 6 months (aOR = 3.314; 95% CI: 1.334–8.232; p = 0.010) and were sexually active for 6–10 years (aOR = 3.076; 95% CI 1.152–8.209; p = 0.025) as compared to their counterparts (Table 1).

### Clinical symptoms and signs

Regarding clinical symptoms and signsrelated to genital infection, the most frequent symptoms reported by study participants were lower abdominal pain (77.6%) followed by vaginal discharge (37.6%), adnexal tenderness (33.2%), urethritis (16.8%), vaginal bleeding (16.1%), cervical excitation (13.0%), dysuria (8.1%) and bilateral masses (5.6%). None of theassessedclinical symptoms was found to be associated with CT infection(Table 2).

**Table 2:** Association between *Chlamydia trachomatis* infection and clinical symptoms among women attending the gynecology clinic of Hawassa University Referral Hospital, 2015.

Clinical symptom	Participants N (%)	CT positive N (%)	COR(95%CI)	p- value
**Lower abdominal pain**				
Yes	250(77.6)	44(17.6)	1	
No	72(24.4)	17(23.6)	1.447(0.768–2.728)	0.253
**Adnexal tenderness**				
Yes	107(33.2)	19(17.1)	1	
No	215(66.8)	42(19.5)	1.124(0.617–2.048)	0.701
**Cervical excitation**				
Yes	42(13.0)	8(19.0)	1.008(0.441–2.302)	0.985
No	280(87)	53(18.9)	1	
**Bilateral masses**				
Yes	18(5.6)	2(11.1)	1	
No	304(94.4)	59(19.4)	1.927(0.431–8.610)	0.391
**Vaginal discharge**				
Yes	121(37.6)	21(17.4)	1	
No	201(62.4)	40(19.9)	1.183(0.660–2.122)	0.573
**Vaginal bleeding**				
Yes	52(16.1)	6(11.5)	1	
No	270(83.9)	55(20.4)	2.012(0.816–4.963)	0.129
**Dysuria**				
Yes	26(8.1)	2(7.7)	1	
No	296(91.9)	59(19.9)	3.090(0.709–13.477)	0.133
**Urethritis**				
Yes	137(42.5)	23(16.8)	1	
No	185(57.5)	38(20.5)	1.078 (0.440–1.384)	

CT, *Chlamydia trachomatis*; N, total number; CI, confidence interval; COR, crude odds ratio.

### Clinical diagnoses

Of the study participants examinedby a gynecologist, 21.1% of the women had a history of PID and 26.4% a history of infertility. Of these, CT infection was found in 20.0% (17/85) of the infertile women and 14.7% of PID patients. Ninety-six (29.8%) of study participants had had an abortion, of whom 45.8% had a history of two or more abortions. None of the clinical diagnoses was found to be significantly associated with CT infection (Table 3).

**Table 3:** *Chlamydia trachomatis* in relation to clinical diagnoses among women attending the gynecology clinic of Hawassa University Referral Hospital, 2015.

Clinical outcomes	Participants N (%)(N = 322)	CT positive N (%)	COR(95%CI)	p- value
**History of abortion**				
Yes	96(29.8)	18(18.8)	1.00(0.543–1.843)	0.999
No	226(70.2)	43(18.9)	1	
**Frequency of abortion**				
One	52(54.2)	9(17.3)	1	
Two and above	44(45.8)	9(20.5)	1.229(0.440–3.428)	0.694
**History of infertility**				
Yes	85(26.4)	17(20.0)	1.097(0.587–2.047)	0.772
No	237(73.6)	44(18.6)	1	
**History of PID**				
Yes	68(21.1)	10(14.7)	1	
No	254(78.9)	51(18.9)	1.457(0.697–3.048)	0.317

CT, *Chlamydia trachomatis*; N, total number; COR, crude odds ratio; PID, pelvic inflammatory disease.

## Discussion

In Ethiopia, laboratory diagnosis of CT infection is not carried out; as a result, urogenital infection due to CT may be considered to be another STI. The actual burden of the disease is not known and management is based on a syndromic approach. Lack of information regarding the prevalence of urogenital CT infection in Ethiopia is also an echo of the absence of specific and rapid diagnostic tests in the country. Thestudy here provides current information on the status of an infection; based on this an intervention strategy could be designed. To the best of our knowledge, this is the first study to provide data on prevalence and risk factors of CT infection in women of reproductive age in Ethiopia.

In thestudy reported here, the overall prevalence of CT antigen among women attending the gynecology clinic was 18.9%. Although the majority of our participants were married and had a single lifetime partner, live in rural areas and did not engage in pre-marital sex, i.e. all low-risk factors for STIs, this finding is comparable with the study done in Nigeria (18.2%) [11], Taiwan (18.4%) [12], Gaza, Palestine (20.2%) [13]and the Solomon Islands (20.3%) [14]. Itis possible that the prevalence of CT infection reported in our study represents a true picture of the disease since the reported condom usage was very low, which may suggest further investigation of other risk behaviors like non-monogamous spouses [15]. In contrast to our finding, higher rates of CT infection were reported in women attending gynecology clinics in Mbarara, Uganda (26.5%) [16], Jos, Nigeria (51.6%) [17]and South Eastern Nigeria(40.7%) [18]. However, lower rates of CT was reported in Western Kenya (3%) [19], North Western Nigeria(9.6%) [15]and Iran (8.3%) [20]. These disparities in prevalence rates could be due to a varying distribution of risk factors in different populationand geographical regions as well as different laboratory methods, which may have different sensitivity and specificity [16, 17].

In this study, a high prevalence of CT infection was reported in the 15–24 age group (24.2%), followed by the 25–44age group (16.8%), showing aninverse relation with age. Similarly, higher infection rates were shown in the 15–24 age group elsewhere [12, 21]. Therefore, the prevalence of CT in adolescent females in this country is an important health concern since untreated CT infection may be a reservoir for ongoing transmission in the population and could have long-term consequences, which may not manifest themselves until women later try to conceive.

Unprotected sex practice puts a woman at risk of both unwanted pregnancy and sexually transmitted infections. In this study, women who practiced unprotected sex within the last six months and were sexually active for 6 to 10 years had about 3.3 times and 3 times higher odds of CT infection, respectively, compared to their counterparts. Similar findings have been reported in other studies [12, 22]. In contrast with previous literature, marital status [15] and other factors that might be associated with CT, including urban residence [16], multiple sexual partners [8], and pre-marital sex were not found to be significant risk factors in this study. In Ethiopia, it was difficult to obtain information regarding sexual behavior because of socio-cultural inhibitions. As a result, participants may have been reluctant to disclose some behaviors, such as multiple lifetime partners and pre-marital sex, which may have limited our ability to detect such associations.

In this study, none of the clinical symptoms and signs correlated with the presence of infection, which suggests that syndromic management alone is unlikely to have a major public health impact in controlling CT infection [23]. This is consistent with previous studies [23–25] that reported the absences of symptom(s) in many women with CT infection. Infected women who are untreated in a timely and effective manner may be at a higher risk of developing gynecological complications [24–26]. A study has shownthat there is a probability of 16% that CT infection will result in PID [7], in line with our finding that 14.7% of CT positive women had a history of PID. In this study, 20% of women with a history of infertility have CT infection concordant with a study in India(18.6%) [27]that showed significant association between current CTinfection andinfertility. Similarly, 20.5% of women who had a history of two or more abortions had CT infection. This needs a further follow-up study to confirm the association of CT infection with infertility and recurrent abortion in Ethiopia.

In this study, we found a low prevalence of *N*. *gonorrhoeae*, which is similar to rates found in other studies [19,28,29]. However, high rates of prevalence were previouslyreported in Ethiopia [30, 31]. The low prevalence rate in our study may be due to difference in the study subjects. In the previous study, only symptomatic cases were enrolled while our study included both symptomatic and asymptomatic cases. In addition, swab transport systems were used in our study in contrast to the study conducted in Northwest Ethiopia [30], which used immediate inoculation to modified Thayer Martin media, which is a method preferred over swab transport systems [32].

The findings in this report have some limitations in light of which results need be interpreted. First, ours was a hospital-based study that used a non-probability sampling method: this may hinder the generalizability of the result to all women of a reproductive age in Ethiopia. Second, due to lack of access, nucleic acid amplification tests (NAATs) was not performed. Despite the above limitations, our study has the strength that CT antigen was detected directly from an endocervical swab using a point-of- care test, indicating active infection in both asymptomatic and symptomatic cases. Moreover, the study may serve as baseline information for further large-scale studies on the general population in the country.

## Conclusion

Relatively high prevalence of CT and low prevalence of gonococcal infection among women of reproductive age wereobserved, especially among those participants who had unprotected sex (no condom use) within the last six months and had been sexually active for 6 to 10 years. Despite its shortcomings, this study provides a strong foundation for the initiation of screening and treatment programs for CT in high-risk populations, particularly among young women aged 15 to 24 years to stem the spread of this infection.
